# The relationship between hormonal levels and hematological parameters in cystic ovarian syndrome

**DOI:** 10.25122/jml-2022-0315

**Published:** 2023-06

**Authors:** Fatema Ali AL kafhage, Ashwaq Najemaldeen Abbas, Rusul Ali Al-Masaoodi, Saif Hassan, Maryam Kadhim Al-Shemery

**Affiliations:** 1 Veterinary Medicine, University of Kerbala, Kerbala, Iraq; 2 Department of Pharmacy, College of Dentistry, University of Sulaymaniyah, Sulaymaniyah, Iraq; 3 College of Applied Medical Sciences, University of Kerbala, Kerbala, Iraq; 4 Department of Pharmacy, Al-Zahrawi University College, Kerbala, Iraq; 5 Faculty of Science, University of Kufa, Kufa, Iraq

**Keywords:** FSH, prolactin, LH, WBC, HB, PLT, FSH: Follicle-Stimulating Hormone, LH: Luteinizing Hormone, PCOS: Polycystic Ovarian Syndrome, IR: Insulin Resistance, LSD: Least Significant Differences

## Abstract

Polycystic ovarian syndrome (PCOS) is characterized by the abnormal production of ovarian androgens resulting in elevated levels of male sex hormones in women. This condition is often marked by the development of a group of small cysts, fluid-filled sacs (cysts) in the ovaries. This study aimed to analyze serum levels of prolactin, follicle-stimulating hormone (FSH), luteinizing hormone (LH), and specific hematological parameters in women with PCOS. In total, 70 women were enrolled, of which 50 were diagnosed with PCOS at an obstetrics institution in Karbala from February and May 2022, and 20 were excluded. Participant selection was based on the Rotterdam 2003 criteria, and we excluded postmenopausal women, those with hyperprolactinemia, and those with overt thyroid dysfunction. The control group included 20 fertile women with normal hormone levels, regular menstrual cycles, and no signs of hyperandrogenism, as verified by ultrasonography. Ages 15 to 46 were similar with regard to the frequency of illness, with those under 36 having a higher incidence. Data were collected via questionnaires, hormone level assessments, and complete blood count (CBC) tests. There was a significant increase in hormone levels (LH, FSH, prolactin, TSH) and CBC values (WBC, Hb, and Plt) in the PCOS group compared to the control group. We observed that women between 26 and 35 were more susceptible to PCOS. Furthermore, women who were overweight demonstrated a higher susceptibility to the syndrome.

## INTRODUCTION

Polycystic ovarian syndrome (PCOS) is a prevalent endocrine disorder that primarily affects women during their reproductive years. It is characterized by irregular menstrual cycles, high levels of androgens, and polycystic ovaries [1]. This condition is regarded as a disease with long-term health effects, including reproductive, metabolic, and cardiovascular aspects affecting between 5% and 10% of infertile women [2]. It is often linked to systemic inflammation and obesity or insulin resistance. PCOS is a multi-system endocrine disorder that poses reproductive and metabolic challenges for women of reproductive age [1-2]. The etiology of PCOS is multifactorial, and while the precise causes are not fully understood, research suggests a combination of genetic, environmental, and dietary factors contribute to its development [3]. Clinical manifestations of PCOS include menstrual irregularities, signs of hyperandrogenism (clinically or through hormone analysis), impaired fertility, polycystic ovaries, and metabolic irregularities such as elevated levels of luteinizing hormone (LH), testosterone, and insulin, along with reduced levels of follicle-stimulating hormone (FSH) [4]. PCOS is characterized by a high amount of androgen and meets the hyperandrogenism criterion. According to the National Institute of Health (NIH) consensus standards, about 80% of women with polycystic ovaries have increased testosterone levels [5]. The signs and symptoms of PCOS, including hirsutism, acne, male pattern baldness, and oligo ovulation (or absence of ovulation), are attributed to hyperandrogenemia. The ovaries, particularly theca cells, and the zona reticularis of the adrenal cortex are the major sources of excessive androgen in PCOS [6]. While the incidence of PCOS can vary due to differences in phenotypic expressions, it remains the most common endocrine disorder among women, with a substantial prevalence [7]. Accurate understanding and management of PCOS are vital to address its complex nature and the diverse challenges it presents in reproductive and metabolic health. This study aimed to analyze serum levels of prolactin, follicle-stimulating hormone (FSH), luteinizing hormone (LH), and specific hematological parameters in women with PCOS.

## MATERIAL AND METHODS

### Study design and location

This cross-sectional study aimed to examine the relationship between serum levels of prolactin, follicle-stimulating hormone (FSH), luteinizing hormone (LH), and specific hematological parameters in women diagnosed with polycystic ovarian syndrome (PCOS). The study was conducted at Al-Husseini Hospital, a tertiary healthcare center in Karbala. Data collection took place between February and May 2022, and participants included a cohort of women aged 18-46 years.

### Participants

The inclusion criteria for this study consisted of women aged 18-46 years who were diagnosed with polycystic ovarian syndrome (PCOS) using the Rotterdam criteria and women who primarily exhibit polycystic ovarian morphology or anovulation (n=50). The study excluded menopausal women (natural or surgical), pregnant or nursing women, individuals with hyperprolactinemia, individuals with overt thyroid dysfunction, and individuals with PCOS Rotterdam criteria who only had one symptom (e.g., hyperandrogenism) (n=20). The two groups made up of the remaining participants were as follows:

1. PCOS group: Females who received a PCOS diagnosis using the Rotterdam criteria.
2. Control: Women who were eumenorrheic, not hirsute, and lacked polycystic ovary morphology (PCOS).

### Study procedures

Participants were scheduled for study procedures on the second or third day of their highly bioavailable or progesterone-induced menstrual cycles. Following an overnight fasting period, venous blood samples were collected from each patient, regardless of their PCOS status, at Al-Husseini Hospital in Holy Karbala. The blood samples were obtained at least two hours after waking up in the morning. All participants underwent either a transvaginal scan or transabdominal ultrasonography of the ovaries using a 3.5-MHz transabdominal and a 5-MHz transvaginal transducer. The ultrasound examinations were conducted on the same day as the blood sample collection to ensure concurrent evaluation. All laboratory measurements were conducted at the Endocrine Research Center. Levels of follicle-stimulating hormone (FSH) and luteinizing hormone (LH) were measured using commercial kits from Izotop (Budapest, Hungary) and analyzed using a Dream Gamma-10 gamma counter from Shin Jin Medics Inc. (Koyang, Korea). Prolactin hormone levels were assessed using diagnostic kits from Diagnostic Biochem Canada Co. (London, ON, Canada). A complete blood count (CDC blood test) was also performed using an Auto Hematology Analyzer in Germany.

### Statistical Analysis

The statistical analysis was conducted using IBM SPSS Statistics software (version 26). To assess the significance of the results, the Least Significant Differences (LSD) test with one-way ANOVA was employed for all data. This test was used to determine the p-value, indicating the level of statistical significance. The Pearson correlation coefficient was also utilized to examine the relationships between the parameters under investigation. A significance level of p<0.05 was used to define statistical significance, indicating that differences or relationships with a p-value below this threshold were considered statistically significant. The data were presented as mean±SD (standard deviation).

## RESULTS

**Figure 1:**
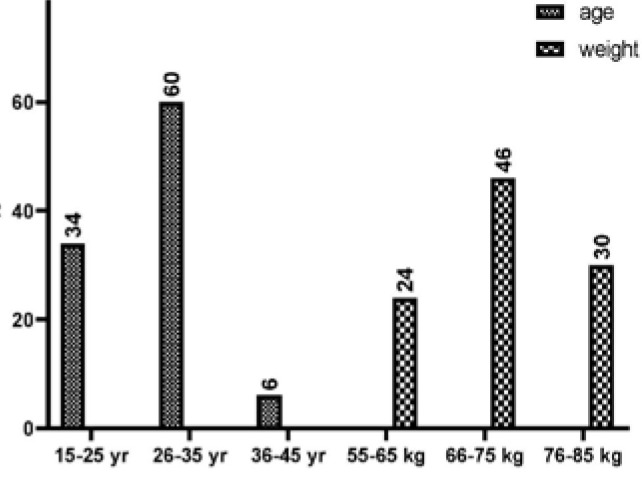
Distribution of sample according to age and weight

The study findings revealed a significant association between age and the prevalence of PCOS among women. Specifically, women aged 26-35 years had a significantly higher percentage of PCOS compared to women in other age groups (p<0.05). Similarly, women in the 66-75 kg weight range showed a higher percentage of PCOS than other weight groups (p< 0.05). These results are summarized in Table 1 and Figure 1.

**Table 1:** Sample distribution according to age and weight

Variables (n=50)	sub.	N	%	p-value
Age	15 – 25	17	34%	0.000
	26 – 35	30	60%	
	36 – 46	3	6%	
	Total	50	100%	
Weight	55 – 65	12	24%	0.020
	66 – 75	15	46%	
	76 – 85	23	30%	
	Total	50	100%	

**Figure 2:**
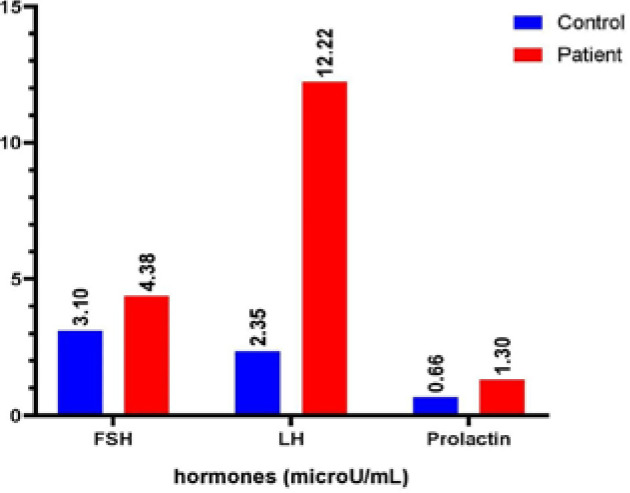
Impact of PCOS on hormone levels

Women with PCOS exhibited significantly higher levels of FSH (4.38±2.05) and LH (12.22±3.31) compared to the control group (FSH: 3.10±1.21; LH: 2.35±1.30) (p<0.05). Additionally, the level of prolactin hormone was significantly elevated in women with PCOS (1.30±0.41) compared to the control group (0.66±0.62) (p<0.05). These results are presented in Table 2 and Figure 2, along with the correlation coefficients between the hormone levels.

**Table 2:** Hormone levels and correlation analysis between control and patient groups (n=50)

Hormones	Group	mean± SD	L.S. D	p-value	r
FSH	Control	3.10±1.21	0.95	0.000	-0.168
	Patient	4.38±2.05			
LH	Control	2.35±1.30	1.47	0.000	-0.885
	Patient	12.22±3.31			
Prolactin	Control	0.66±0.62	0.24	0.000	-0.821
	Patient	1.30±0.41			

### Effect of polycystic ovary syndrome (PCOS) on hematology

**Figure 3:**
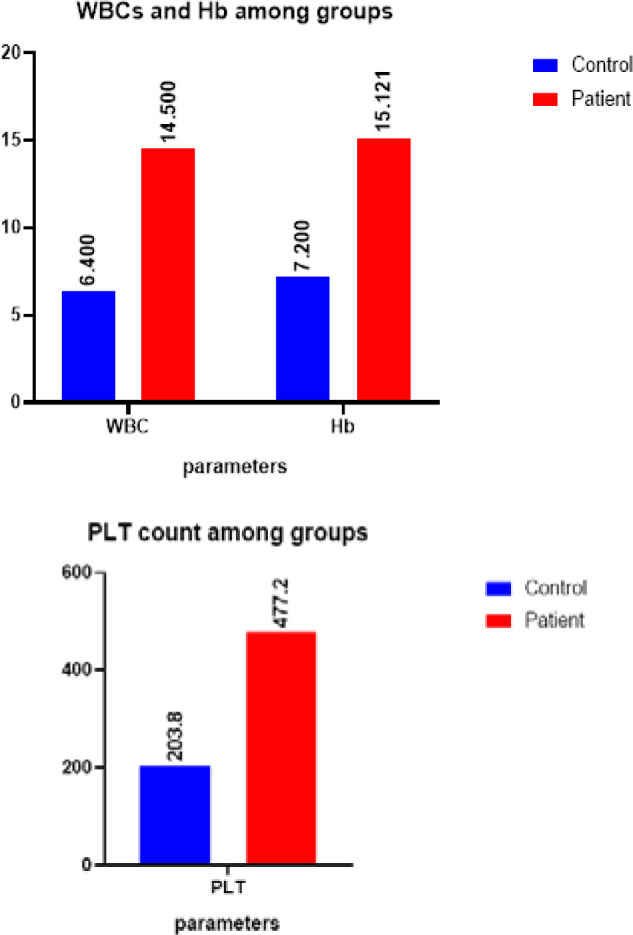
Effect of polycystic ovary syndrome (PCOS) on hematology

Regarding hematology, women with PCOS demonstrated significantly higher levels of white blood cells (WBC), platelets (PLT), and hemoglobin (Hb) compared to the control group (p<0.05). The mean values for WBC, PLT, and Hb were higher in the PCOS group (WBC: 14.50±1.97; PLT: 477.2±111.4; Hb: 15.121.14) compared to the control group (WBC: 6.4±2.3; PLT: 203.8±69.09; Hb: 7.2±1.93) (Table 3, Figure 3).

**Table 3:** The level and correlation of CBC results in women with PCOS and its comparison with the control group (n=50)

	Group	mean± SD	L.S. D	p-value	r
WBC	Control	6.4±2.3	1.09	0.000	-0.992
	Patient	14.50±1.97			
PLT	Control	203.8±69.09	52.16	0.000	-0.932
	Patient	477.2±111.4			
HB	Control	7.2± .93	0.748	0.000	-0.532
	Patient	15.121.14			

## DISCUSSION

The study findings revealed that overweight women are at a higher risk of developing polycystic ovarian syndrome (PCOS), as observed in Table 2. This result aligns with previous research [8], which also reported a significant prevalence of obesity among women with PCOS. These findings support the notion that obesity plays a role in the development and manifestation of PCOS. Obesity is a prevalent feature in PCOS patients [9]. Obesity might contribute to the development of PCOS in susceptible individuals due to the hormonal effects of adipose tissue. Adipose tissue, known for its endocrine function, plays a role in the hormonal dysregulation observed in PCOS [10]. The association between obesity and PCOS is further supported by the observed menstrual irregularities and hyperandrogenic status in affected women. The irregular menstrual cycles seen in PCOS, such as oligomenorrhea and amenorrhea, can be attributed to anovulation [11]. Anovulation results in the formation of no corpus luteum, which prevents the release of progesterone, which makes the proliferative phase excessively active [12]. The GnRH pulse frequency indicates the predominant release of LH through high-frequency pulses compared to FSH through low-frequency pulses in healthy adult women. Progesterone plays a role in modulating the pulse frequency of gonadotropin-releasing hormone (GnRH) in the presence of estradiol. Increased progesterone production by the corpus luteum leads to a reduction in the frequency of LH (luteinizing hormone) pulses.

On the other hand, FSH (follicle-stimulating hormone) production is favored as it supports the expansion of follicles for the upcoming menstrual cycle [13]. The source of LH (luteinizing hormone) hypersecretion in PCOS (polycystic ovary syndrome) is more likely attributed to greater pituitary sensitivity to GnRH (gonadotropin-releasing hormone) or alterations in GnRH secretion patterns rather than increased GnRH secretion itself. This hypersecretion of LH appears to be a result of impaired regulation. In PCOS, the pituitary and hypothalamus exhibit reduced susceptibility to the inhibitory effects of exogenous progesterone on LH secretion, and there is a lack of cyclic progesterone synthesis by a corpus luteum [14]. Changes in LH secretion patterns could be caused by altered sex steroid production, metabolic malfunction, or obesity. The rise in the duration of interleukin is due to a considerable increase in the total and progressive pain numerical census. Hair filtration at the back of the head is increased by phagocytic cells [15]. Persistent thrombocytosis may exacerbate the preexisting procoagulant condition in PCOS caused by coagulation cascade stimulation, platelet activation, and endothelial dysfunction. The study analyzed various factors related to cardiovascular risk in women with PCOS, including traditional cardiovascular risk variables and certain indicators associated with proinflammatory and procoagulant tendencies. Hemoglobin concentrations may be affected by oligomenorrhea or amenorrhea, androgen excess, two important diagnostic criteria for PCOS. In comparison with healthy controls, women with PCOS had significantly greater hemoglobin levels and a decreased incidence of anemia, according to the findings [16].

## CONCLUSION

Our findings indicate that women between the ages of 26 and 35 were more susceptible to PCOS, highlighting the importance of this age group in terms of risk assessment and preventive measures. Additionally, the study confirms the significant impact of obesity on the prevalence of PCOS, with overweight women showing a higher susceptibility to the syndrome.
